# Positive feedback of the amphiregulin-EGFR-ERK pathway mediates PM2.5 from wood smoke-induced MUC5AC expression in epithelial cells

**DOI:** 10.1038/s41598-017-11541-1

**Published:** 2017-09-11

**Authors:** Lingmei Huang, Jinding Pu, Fang He, Baoling Liao, Binwei Hao, Wei Hong, Xiuqin Ye, Jinglong Chen, Jun Zhao, Sha Liu, Juan Xu, Bing Li, Pixin Ran

**Affiliations:** 1 State Key Laboratory of Respiratory Disease, The First Affiliated Hospital of Guangzhou Medical University, Guangzhou, Guangdong, China; 2 School of Basic Medical Sciences, Guangzhou Medical University, Guangzhou, Guangdong China; 3 GMU-GIBH Joint School of Life Sciences, Guangzhou Medical University, Guangzhou, Guangdong China; 4 The First People’s Hospital of YueYang, Yueyang, Hunan China; 5 Guangzhou First People’s Hospital, Guangzhou medical university, Guangzhou, Guangdong China

## Abstract

Biomass fuel smoke is thought to contribute to chronic obstructive pulmonary disease, which is characterized by mucous cell metaplasia and enhanced mucus secretion. We investigated the effect of particulate matter (PM) with a diameter <2.5 μm (PM2.5) from wood smoke (WSPM2.5) on the expression of the most prominent secreted mucin, MUC5AC. Wood smoke was able to induce MUC5AC expression in the rat respiratory tract after 3 months of exposure. WSPM2.5 could induce MUC5AC production in both primary human airway epithelial cells and the NCI-H292 cell line. This induction process was mediated by activation of epithelial growth factor receptor (EGFR)-extracellular signal-regulated kinase (ERK) signaling through an EGFR ligand-dependent mechanism. Amphiregulin (AR) was identified as the major ligand responsible for EGFR-ERK signaling activation and MUC5AC expression. In turn, EGFR-ERK pathway activation was found to contribute to the *de novo* synthesis of AR. This positive feedback loop might play an important role in a sustained mucus hypersecretion response.

## Introduction

Chronic obstructive pulmonary disease (COPD), a major increasing global public health problem, is estimated to become the world’s third leading cause of death by 2020^1^. Ambient air pollution, including wood smoke pollution, is suspected to be a contributor to an increased risk of COPD^2, 3^. Because more than half the world’s population – approximately 3 billion people – are exposed to biomass smoke, this type of smoke, rather than tobacco smoke, appears to be the greatest risk factor for COPD^4^. Patients exposed to biomass smoke present with an airway-predominant phenotype, which differs from the emphysema-predominant phenotype observed in tobacco-related COPD^5^. Hence, the detailed mechanisms responsible for wood smoke-induced COPD need to be fully elucidated to develop specific therapeutic approaches.

Mucus hypersecretion is a major manifestation of COPD and contributes to airway obstruction, lower respiratory infection, accelerated lung function decline, morbidity and mortality^6–11^. MUC5AC and MUC5B are the most abundant secreted mucins, and MUC5AC secretion is considered the central event in mucous metaplasia^12^.

Particulate matter (PM), a major component in wood smoke^13^, has been thought to be the single most important indicator of the health effects of wood combustion sources. However, unlike other fossil fuel combustion chemicals, the health effects of PM from wood smoke remain poorly studied. Due to their different composition, the wood smoke particles may not produce the same health impacts as other particles^14, 15^. Urban PM with an aerodynamic diameter <2.5 μm (PM2.5) can induce airway MUC5AC expression^16^. Hence, whether PM2.5 from wood smoke (WSPM2.5) possesses the same capacity to induce MUC5AC expression must be determined.

Epidermal growth factor receptor (EGFR)-activated extracellular signal-regulated kinase (ERK) signaling plays a critical role in MUC5AC induction^17–19^. EGFR and its ligands are overexpressed in the airway epithelia of COPD patients^20^. Thus, EGFR and its ligands likely participate in mucin induction in response to WSPM2.5 exposure.

We recently provided direct evidence that chronic exposure to biomass fuel induces pulmonary changes in rats consistent with those observed in COPD lungs, including airway mucus hypersecretion^21^. These observations led us to investigate, whether the cellular responses to WSPM2.5 involved MUC5AC secretion, focusing on the EGFR-ERK pathway. Here, we show that WSPM2.5 up-regulates MUC5AC production, that the activation of EGFR-ERK signaling via the autocrine effects of the EGFR ligand AR and secreted AR further contribute to AR expression in a positive feedback loop, and that the EGFR-ERK pathway plays an indispensable role in this process.

## Results

### Wood smoke induces goblet cell hyperplasia and MUC5AC overproduction in rats

**Figure 1:**
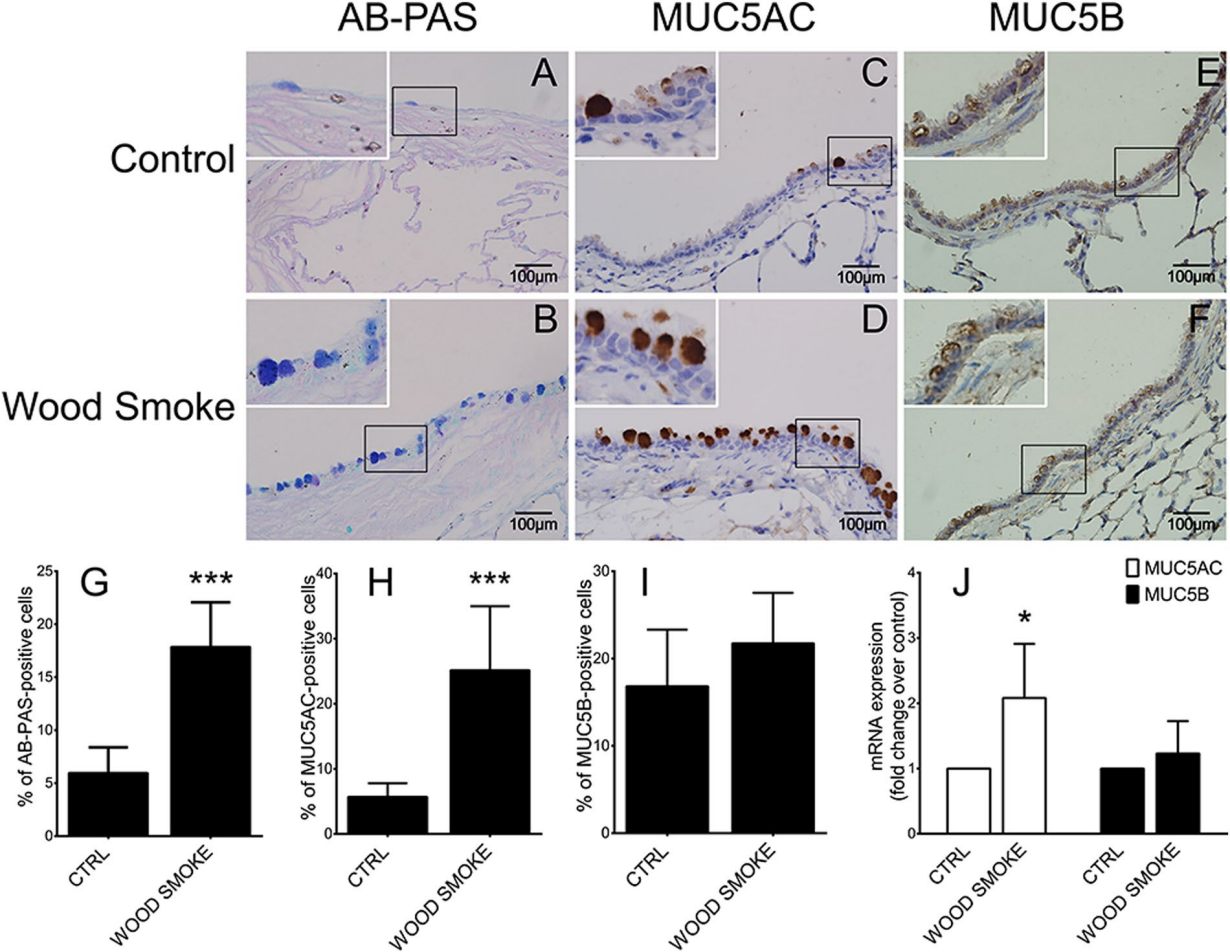
Wood smoke-induced airway mucous cell metaplasia and MUC5AC hyperproduction in the rat airway. Lung sections from rats exposed to control air or wood smoke were stained with AB-PAS (**A**,**B**) or a DAB staining kit using anti-MUC5AC (**C,D**) or anti-MUC5B primary antibodies (**E**,**F**). All images are representative of the results from five independent experiments. (**G**) AB-PAS-positive airway epithelial cells (images **A** and **B**). (**H**) MUC5AC-positive airway epithelial cells (images **C**,**D**). (**I**) MUC5B-positive airway epithelial cells (images **E** and **F**). (**J**) MUC5AC and MUC5B expression in whole lung, as determined by real-time PCR. CTRL: air control group; WOOD SMOKE: wood smoke-treated group. The data are presented as the means ± SD; n = 5. *P < 0.05; ***P < 0.001. Scale bar: 100 μm.

As shown in Fig. 1, exposure to wood smoke for 3 months induced severe airway mucous cell metaplasia, whereas the control animals did not exhibit mucous cell metaplasia (Fig. 1A,B and G). Furthermore, both immunochemistry (Fig. 1C,D and H) and real-time PCR (Fig. 1J) revealed that MUC5AC levels were highly elevated in the wood smoke group. MUC5B expression (Fig. 1E,F,I and J) was also elevated after wood smoke challenge, although this changes was not statistically significant. Thus, wood smoke induced significant mucous cell metaplasia and MUC5AC production in the rat airway.

### WSPM2.5 induces MUC5AC expression in primary epithelial cells cultured at the air-liquid interface

**Figure 2:**
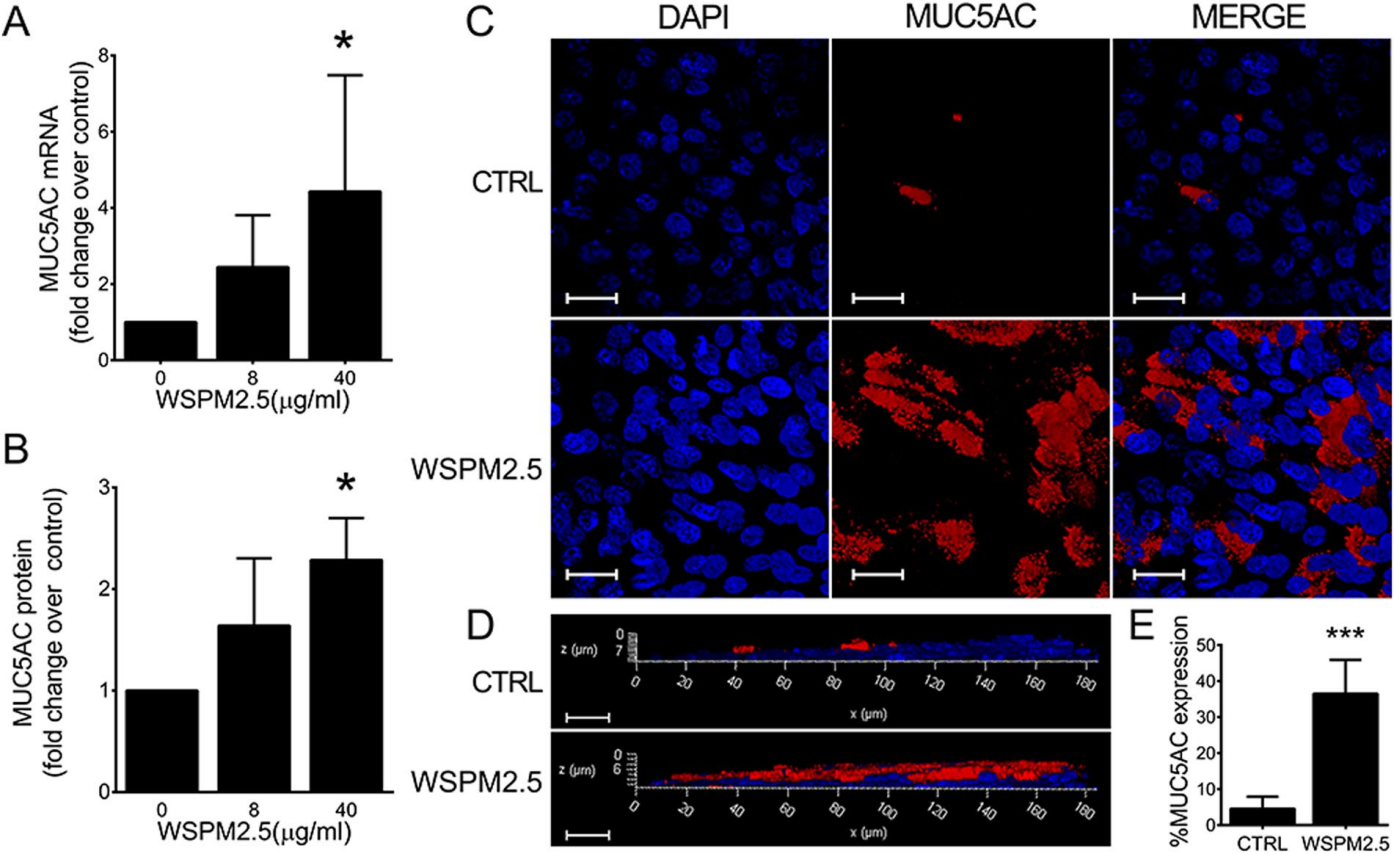
WSPM2.5 induces MUC5AC expression in primary epithelial cells cultured at the air-liquid interface. (**A**,**B**) Epithelial cells were treated apically with WSPM2.5 (8 or 40 μg/ml) for 24 h, and the relative MUC5AC mRNA expression levels were detected by real-time PCR (**A**), while the MUC5AC protein expression levels in the culture supernatants were measured by ELISA (**B**). (**C**,**D** and **E**) Differentiated epithelial cells were treated with WSPM2.5 (40 μg/ml) for 24 h. The filters were cut, stained with anti-MUC5AC antibodies and visualized with a Cy3-conjugated secondary antibody (red, MUC5AC). Nuclei were stained with DAPI (blue). (**C**) Top-down images of stained cells. (**D**) Z-stack images of stained cells. All images are representative of three independent experiments. (**E**) MUC5AC induction was quantified by determining the ratio of MUC5AC fluorescence signal to total DAPI fluorescence using Image-Pro-Plus 6.0 software. The data are presented as the means ± SD. n = 3. *P < 0.05; ***P < 0.001. Scale bar: 20 μm.

Because *in vivo* experiments showed that MUC5AC, but not MUC5B, was highly induced by wood smoke, we next investigated whether WSPM2.5 could induce MUC5AC expression in primary epithelial cells cultured at the air-liquid interface. MUC5AC mRNA expression increased 2.44- and 4.55-fold (Fig. 2A), whereas MUC5AC protein expression increased 1.64- and 2.29-fold in the presence of 8 and 40 μg/ml WSPM2.5, respectively (Fig. 2B). Immunofluorescence staining also indicated that 40 μg/ml WSPM2.5 induced MUC5AC expression (red color in Fig. 2C,D and E).

### WSPM2.5 induces MUC5AC expression in NCI-H292 cells

**Figure 3:**
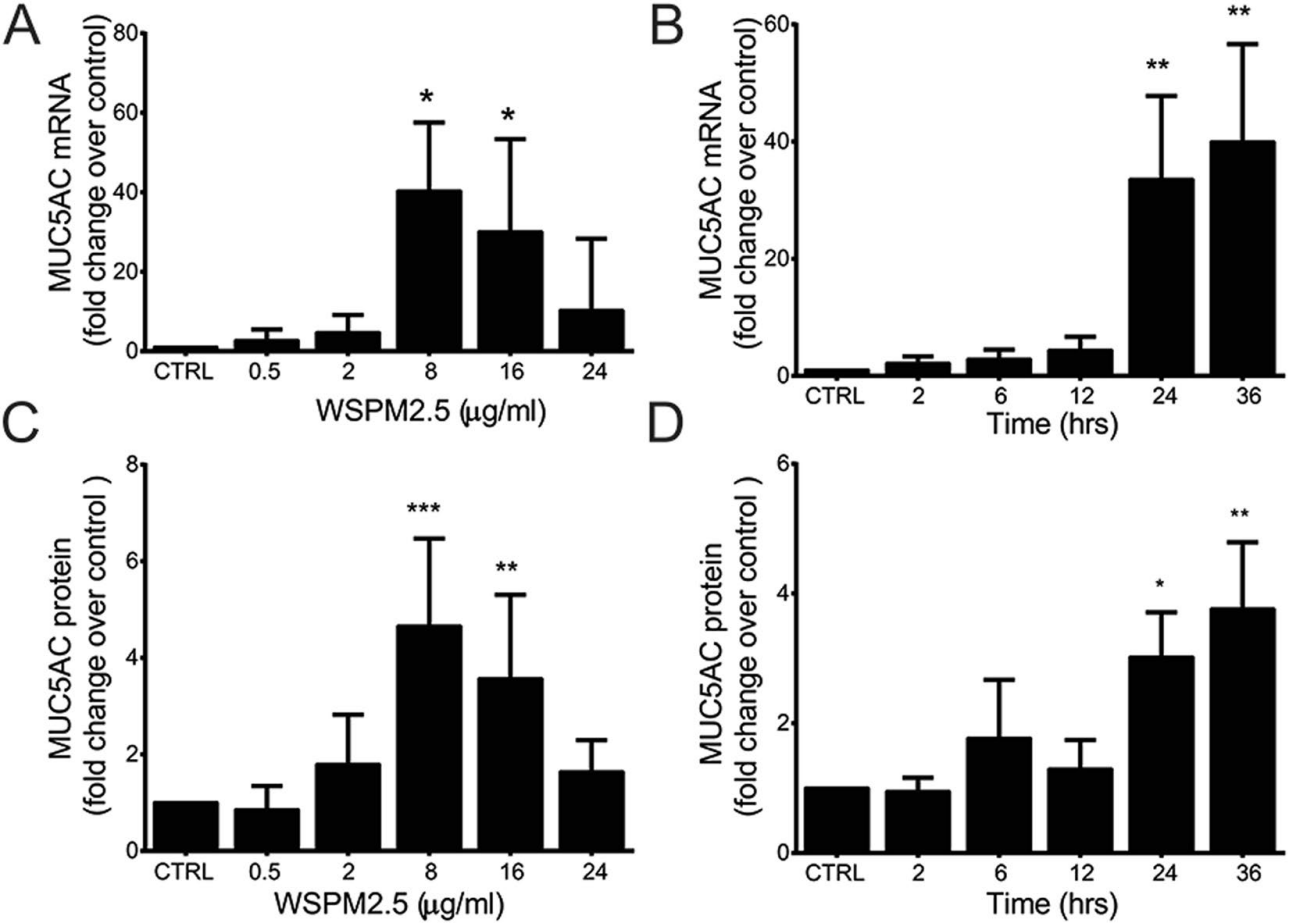
WSPM2.5 induces MUC5AC expression in NCI-H292 cells. Cells were treated with non-cytotoxic concentrations of WSPM2.5 (0.5–24 μg/ml) (**A** and **C**) for 24 h or were treated with WSPM2.5 (8 μg/ml) for various times (2–36 h) (**B** and **D**). MUC5AC mRNA expression levels were determined with real-time PCR, and MUC5AC protein expression levels in the culture supernatants were measured by ELISA. CTRL: WSPM2.5-untreated cells. The values are expressed as the means ± SD; n = 5 (**A**), n = 3 (**B** and **D**) or n = 6 (**C**). *P < 0.05; **P < 0.01; ***P < 0.001. For (**A–D**), the fold increase is the relative change over untreated cells at each concentration or time point.

In the NCI-H292 epithelial cell line, WSPM2.5 dose dependently increased MUC5AC mRNA (Fig. 3A) and protein expression levels (Fig. 3C) (at 8 μg/ml, WSPM2.5 increased MUC5AC mRNA expression 40.26-fold and MUC5AC protein expression 4.65-fold). WSPM2.5 also induced MUC5AC production time-dependently [both MUC5AC mRNA (Fig. 3B) and MUC5AC protein levels (Fig. 3D) increased continuously throughout the 36-h experiment (WSPM2.5, 8 μg/ml)].

### Ligand-dependent EGFR activation mediates WSPM2.5-induced MUC5AC expression

**Figure 4:**
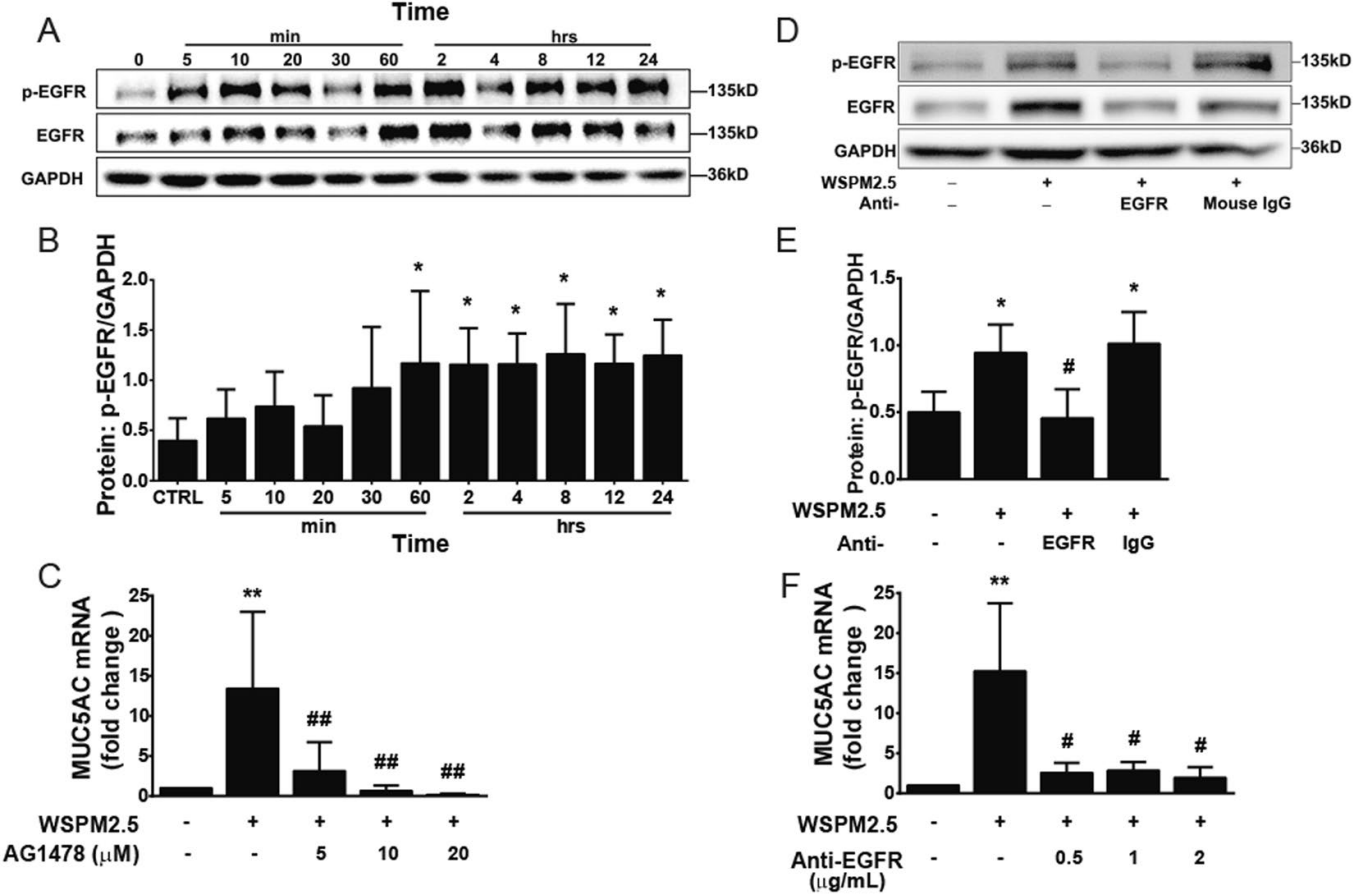
Ligand-dependent EGFR activation mediates MUC5AC induction by WSPM2.5. (**A**) NCI-H292 cells were stimulated with WSPM2.5 (8 μg/ml) for the indicated times. Cell lysates were prepared for Western blot analysis. Antibodies specific for EGFR, phosphorylated EGFR (p-EGFR) and GAPDH were used. (**B**) Density quantification of EGFR phosphorylation in (**A**). (**C**) NCI-H292 cells were pretreated with or without AG1478 (5, 10, or 20 μM) for 1 h and were then treated with WSPM2.5 for 24 h to measure MUC5AC mRNA expression. (**D**) NCI-H292 cells were pretreated with or without an EGFR-neutralizing antibody or a mouse control IgG antibody (0.5 μg/ml) for 1 h and were then stimulated with WSPM2.5 (8 μg/ml) for 60 min. Cell lysates were collected to examine EGFR phosphorylation. (**E**) Density quantification of EGFR phosphorylation in (**D**). (**F**) NCI-H292 cells were pretreated with or without different concentrations of EGFR-neutralizing antibody (0.5, 1, or 2 μg/ml) for 1 h before treatment with WSPM2.5 (8 μg/ml) for 24 h to analyze the MUC5AC mRNA expression levels. (**A** and **D**) one representative gel is shown. Densitometry analysis of p-EGFR was performed after normalization to GAPDH. The data are expressed as the means ± SD. n = 5 (**A,B** and **C**) or n = 4 (**D**,**E** and **F**). *P < 0.05, **P < 0.01 compared with the control; ^#^ *P* < 0.05, ^##^ *P < *0.01 compared with the WSPM2.5 group.

Because MUC5AC production is highly regulated at the transcriptional level^22^, we focused on the mechanism through which WSPM2.5 mediates MUC5AC mRNA induction. EGFR phosphorylation increased continuously, and a significant increase began to be observed at 60 min (Fig. 4A and B). Pretreatment with AG1478, an EGFR-selective tyrosine kinase inhibitor, significantly prevented MUC5AC expression (Fig. 4C). In addition, treatment with EGFR-neutralizing antibody, which prevents ligand binding to EGFR, significantly reduced EGFR phosphorylation (Fig. 4D and E) and MUC5AC expression (Fig. 4F), suggesting ligand-dependent EGFR activation in WSPM2.5-mediated MUC5AC expression.

### EGFR-dependent ERK signaling is involved in WSPM2.5-induced MUC5AC expression

**Figure 5:**
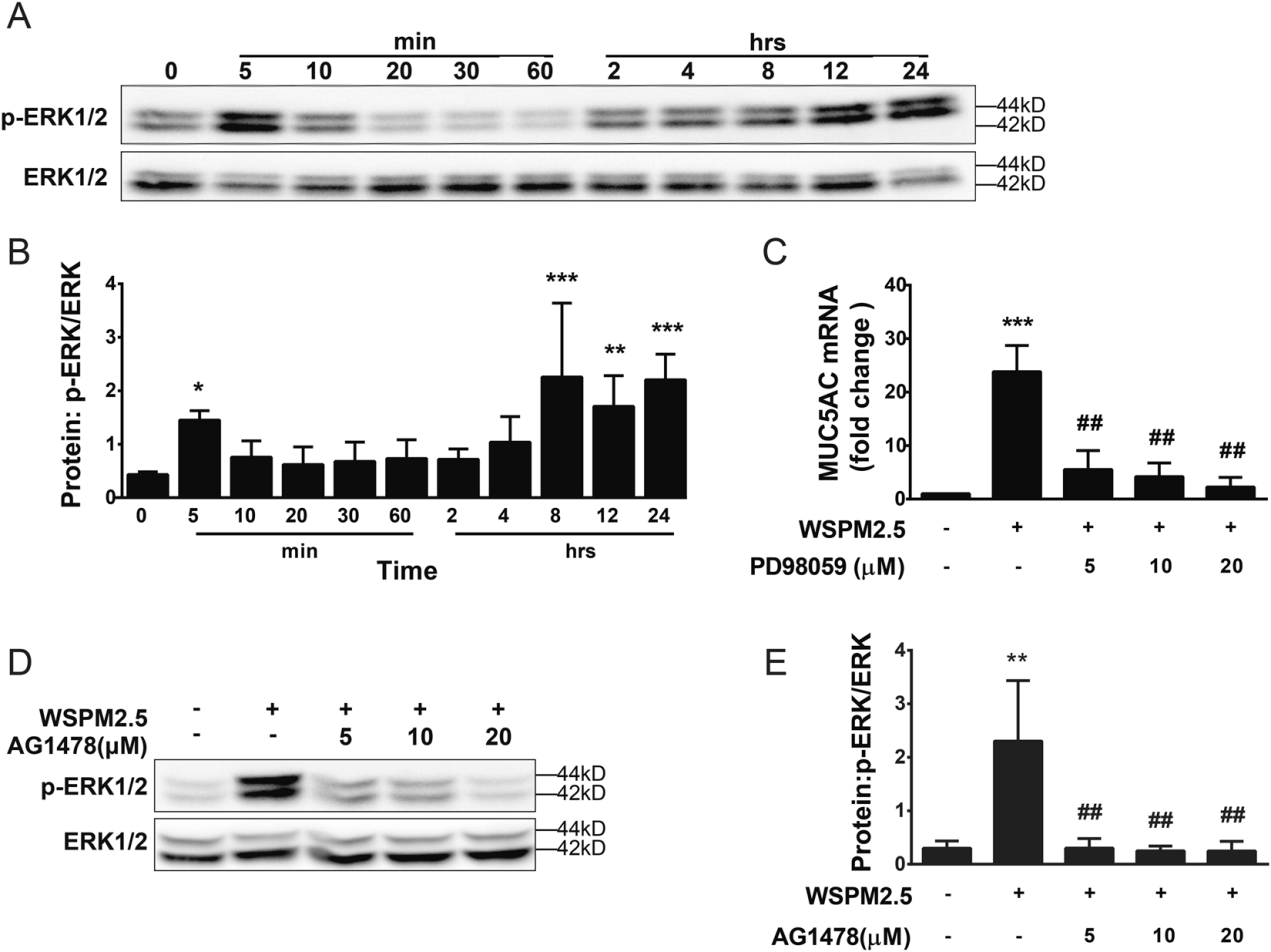
WSPM2.5-induced MUC5AC expression is mediated through the EGFR-dependent ERK pathway. (**A**) NCI-H292 cells were stimulated with WSPM2.5 for the indicated durations. Cell lysates were collected and assayed for ERK phosphorylation. The membranes were stripped and re-probed with an anti-ERK antibody. (**B**) Density quantification of ERK phosphorylation in (**A**). (**C**) NCI-H292 cells were pretreated with or without PD98059 (5, 10, or 20 µM) for 1 h and were then treated with WSPM2.5 for 24 h. MUC5AC mRNA expression was determined by real-time PCR. (**D**) NCI-H292 cells were pretreated with or without AG1478 (5, 10, or 20 μM) for 1 h and were then stimulated with WSPM2.5 for 5 min to assay for ERK phosphorylation. (**E**) Density quantification of ERK phosphorylation in (**D**). (**A** and **D**) one representative gel from three independent experiments is shown. The data are expressed as the means ± SD (n = 3). *P < 0.05, **P < 0.01, ***P < 0.001 compared with the control; ^##^ *P < *0.01 compared with the WSPM2.5 group.

As shown in Fig. 5A and B, biphasic activation of ERK was observed, with phosphorylation starting within 5 min. The second wave started at 8 h and lasted until 24 h. Furthermore, pretreatment with the selective ERK kinase inhibitor PD98059 (Fig. 5C) significantly inhibited WSPM2.5-induced MUC5AC expression, indicating that WSPM2.5-induced MUC5AC production is dependent on ERK activation. EGFR appears to function upstream of ERK, as AG1478 abolished ERK activation (Fig. 5D and E).

### Impact of WSPM2.5 on EGFR ligand expression

**Figure 6:**
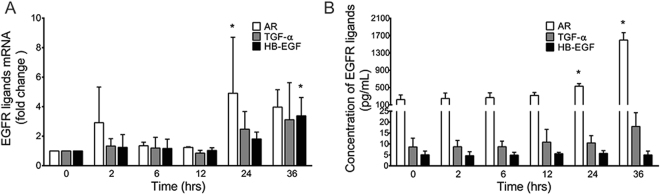
Expression and release of the EGFR ligands AR, TGF-α and HB-EGF in response to WSPM2.5. NCI-H292 cells were exposed to 8 μg/ml WSPM2.5 for 2–36 h, after which RNA was extracted to determine AR, TGF-α and HB-EGF mRNA expression (**A**), or cell culture medium was collected to assess protein expression with ELISA (**B**). The data are expressed as the means ± SD; n = 3. *P < 0.05 compared with the control.

Several EGFR ligands, including AR, transforming growth factor (TGF)-α, and heparin-binding EGF-like growth factor (HB-EGF), have been documented in epithelial mucin regulation^23–25^. The time-course study of WSPM2.5 exposure revealed elevated AR mRNA levels at 2 h (2.91-fold), with a significant increase at 24 h. Although the TGF-α mRNA levels increased, this increase failed to reach significance. HB-EGF mRNA expression was significantly higher after 36 h of exposure (3.38-fold; Fig. 6A). Consistent with the increase in AR mRNA expression, the AR protein levels were significantly higher at 24 and 36 h. WSPM2.5 induced a slight increase in TGF-α release. Inconsistent with the mRNA results, HB-EGF expression remained unchanged (Fig. 6B).

### AR is the primary ligand responsible for EGFR-ERK activation and MUC5AC induction

**Figure 7:**
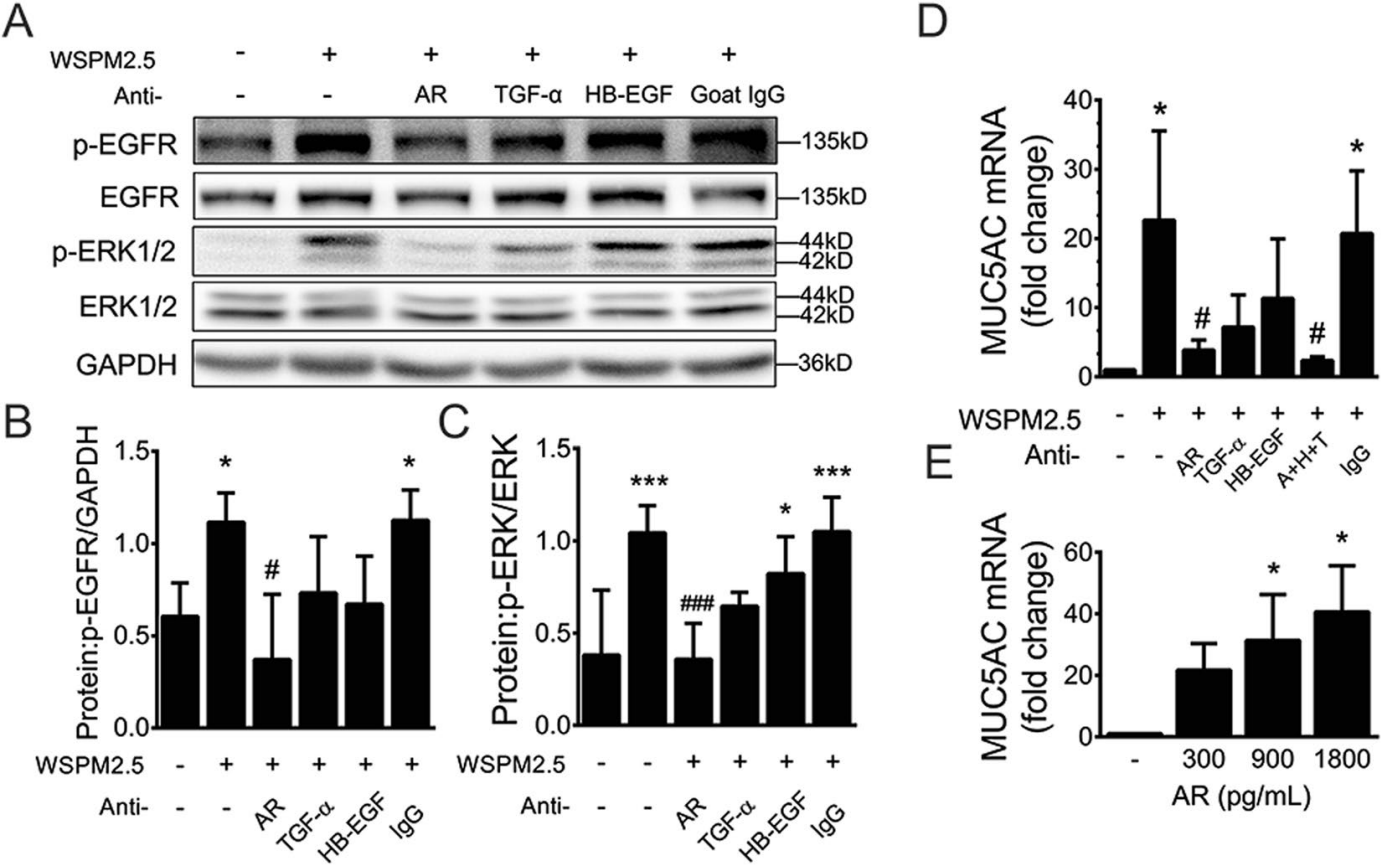
AR is the primary ligand responsible for the activation of EGFR-ERK signaling and MUC5AC expression. Cells were pretreated with neutralizing antibodies against AR, TGF-α or HB-EGF for 1 h and were then stimulated with WSPM2.5 for 60 or 5 min to assay for phosphorylation of EGFR and ERK, respectively (**A**), or for 24 h to measure MUC5AC mRNA expression (**D**). (**B**) Density quantification of EGFR phosphorylation, and (**C**) density quantification of ERK phosphorylation in (**A**). (**E**) NCI-H292 cells were exposed to recombinant AR (300–1,800 pg/ml) for 24 h to assess MUC5AC mRNA expression. (**A**) one representative gel from three independent experiments is shown. Densitometry analysis of p-EGFR and p-ERK after normalization to GAPDH or total ERK, respectively. The data are expressed as the means ± SD; n = 3. *P < 0.05, ***P < 0.001 compared with the control; ^#^ *P* < 0.05, ^###^ *P < *0.001 compared with WSPM2.5- treated cells.

Next, we used specific neutralizing antibodies to prevent ligands from binding to EGFR. Interestingly, AR neutralization suppressed most of the WSPM2.5-induced EGFR and ERK phosphorylation (Fig. 7A,B and C) and MUC5AC gene expression (Fig. 7D), whereas blockade of TGF-α or HB-EGF showed a partial reduction, but this decrease failed to reach statistical significance. The combination of all three antibodies (A + H + T) suppressed MUC5AC expression more than the AR-neutralizing antibody alone. Furthermore, recombinant AR dose-dependently induced MUC5AC expression (Fig. 7E). Thus, AR is crucial in mediating the WSPM2.5-induced activation of the EGFR-ERK pathway and MUC5AC up-regulation.

### The *de novo* synthesis of AR contributes to MUC5AC transcriptional regulation

**Figure 8:**
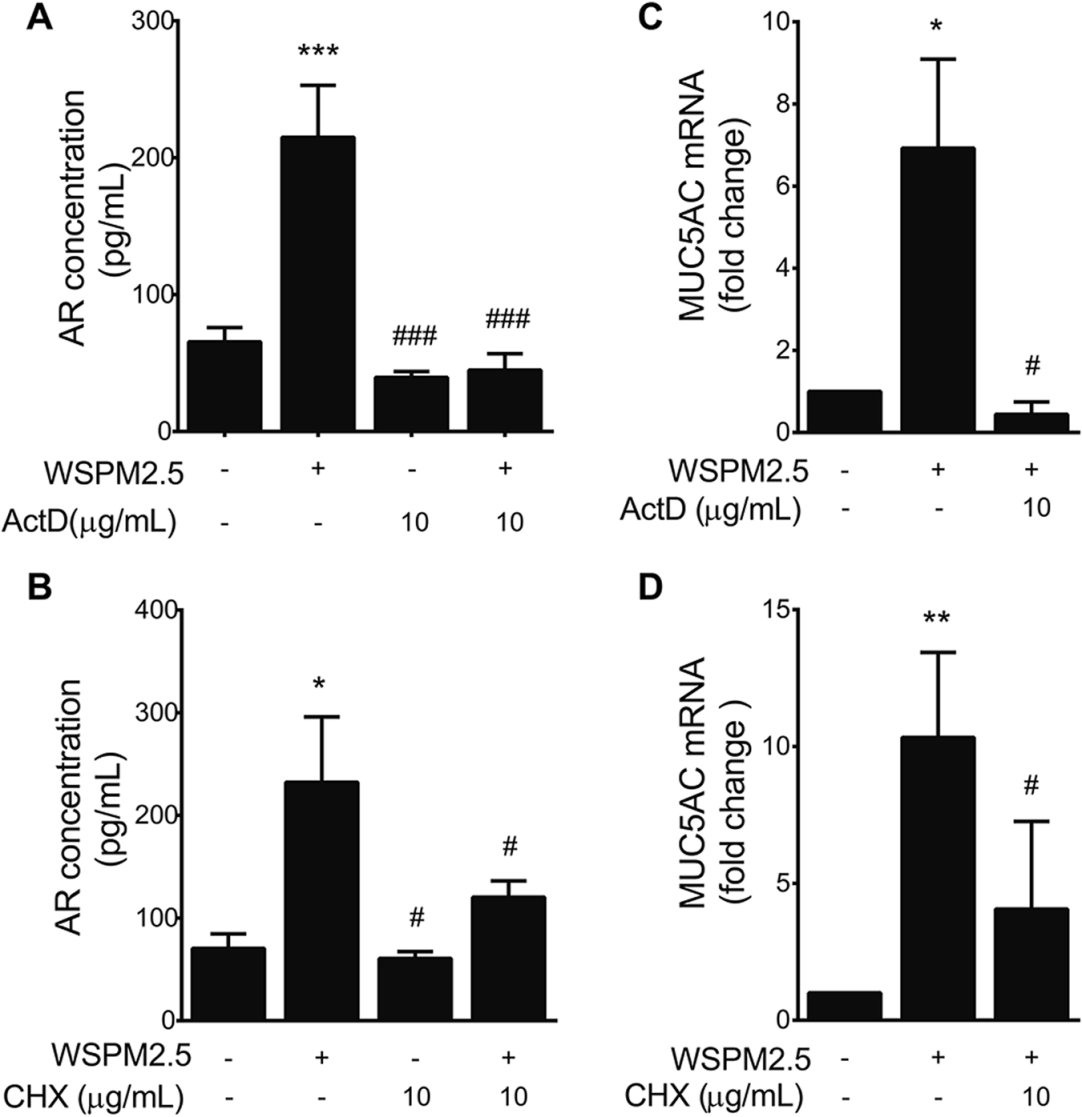
Effects of *de novo* AR synthesis on WSPM2.5-induced MUC5AC mRNA expression. NCI-H292 cells were pretreated with ActD (10 μg/ml) (**A** and **C**) or CHX (10 μg/ml) (**B** and **D**) followed by WSPM2.5 exposure for 24 h. AR protein expression was assessed by ELISA (**A** and **B**), and MUC5AC mRNA expression was determined by real-time PCR (**C** and **D**). The data are expressed as the means ± SD; n = 3. *P < 0.05, **P < 0.01, ***P < 0.001 compared with the control; ^#^ *P* < 0.05, ^###^ *P* < 0.001 compared with WSPM2.5-treated cells.

To assess whether increased AR expression occurred due to increased *de novo* protein synthesis, we treated NCI-H292 cells with actinomycin D (ActD) and cycloheximide (CHX), known inhibitors of transcription and *de novo* protein synthesis, respectively. ActD and CHX not only significantly inhibited AR secretion at baseline or upon WSPM2.5 stimulation (Fig. 8A and B) but also attenuated MUC5AC mRNA expression (Fig. 8C and D), indicating that *de novo* synthesis of AR is needed for the transcriptional regulation of MUC5AC.

### The autocrine effects of AR prolong WSPM2.5-induced AR gene expression and are mediated by the EGFR-ERK pathway

**Figure 9:**
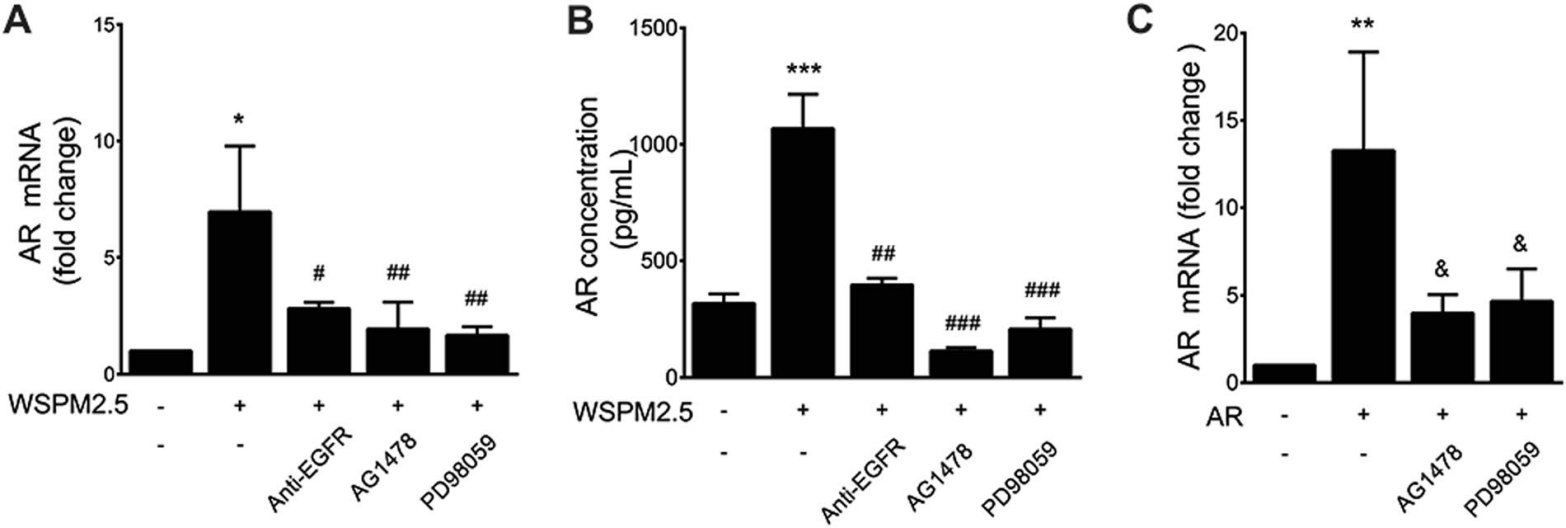
The EGFR-ERK pathway is involved in WSPM2.5-induced AR expression. NCI-H292 cells were pretreated with or without EGFR-neutralizing antibody (0.5 μg/ml), AG1478 (10 μM) or PD98059 (5 μM) for 1 h and were then stimulated with WSPM2.5 for 24 h. AR mRNA was measured by real-time PCR (**A**), and AR release was determined by ELISA (**B**). (**C**) NCI-H292 cells were exposed to 1,800 pg/ml AR with or without AG1478 (10 μM) or PD98059 (5 μM), and AR mRNA expression was analyzed. The data are expressed as the means ± SD; n = 3. *P < 0.05, **P < 0.01, ***P < 0.001 compared with the control; ^#^ *P* < 0.05, ^##^ *P < *0.01, ^###^ *P* < 0.001 compared with the WSPM2.5 group. ^&^ *P* < 0.05 compared with the AR-treated group.

We next sought to clarify the mechanism mediating the autocrine effects of AR. Pretreatment with anti-EGFR, AG1478 or PD98059 significantly reduced WSPM2.5-induced AR mRNA expression (Fig. 9A) and AR release (Fig. 9B), indicating that the autocrine effects of AR are mediated by the EGFR-ERK pathway in epithelial cells. Furthermore, treatment with exogenous AR (1800 pg/ml) increased AR mRNA expression, an effect that was inhibited in the presence of AG1478 or PD98059 (Fig. 9C), suggesting that the prolonged effect of WSPM2.5 on AR mRNA is dependent on the autocrine stimulation of AR through the EGFR-ERK pathway.

## Discussion

In this study, we showed that wood smoke induces mucus production and MUC5AC expression in the rat respiratory tract. We further confirmed that WSPM2.5 induces MUC5AC expression in both primary human airway epithelial cells and NCI-H292 cells. Because mucus hypersecretion is an independent and statistically significant predictor of COPD^26^, we believe that mucus overproduction contributes significantly to the morbidity of wood smoke exposure-induced COPD. Therefore, we set up experiments to explore the molecular mechanisms through which WSPM2.5 affects MUC5AC expression. Our study showed that WSPM2.5 induces MUC5AC via the AR-EGFR-ERK pathway. Interestingly, EGFR-ERK pathway activation further contributes to the autocrine effect of AR expression. Thus, this positive feedback loop between the autocrine effect of WSPM2.5-induced AR release and EGFR-ERK activation facilitates sustained mucus hypersecretion.

Our results confirm that the EGFR-ERK pathway is activated predominantly in a ligand-dependent manner and plays a pivotal role in mucus production, leaving the question of how this receptor is activated during WSPM2.5 exposure to be answered. Because the initial phosphorylation of EGFR-ERK was inhibited by AR-neutralizing antibody, AR is thought to be the critical driver of EGFR-ERK-mediated MUC5AC induction. Notably, AR levels increased significantly after 24 h of treatment, an increase that coincided with mucin production, whereas EGFR phosphorylation occurred after 5 min of treatment. A more reasonable explanation is that the triggering of this pathway does not occur at the ligand level, but rather as a more instant event that initiates the synthesis and release of AR or other ligands. Continuous stimulation by PM then leads to ligand accumulation in the medium and finally causes initiation of mucin production and release into the respiratory tract. The initiating events may occur as follows. First, the rapid increase in AR protein release could result from the proteolytic cleavage of tumor necrosis factor-alpha converting enzyme (TACE), one of the most important metalloproteases that has been reported to play a critical role in mucin production in response to multiple stimuli^27, 28^. We attempted to identify the transient increase in AR release in cell culture; however, it was challenging to obtain consistent results. In addition, reactive oxygen species might also play important roles in EGFR and ERK activation because EGFR-ERK phosphorylation was also reduced by the antioxidant N-acetyl-L-cysteine (NAC) (Supplementary Fig. 1 **)**. Additional studies are needed to clarify this result.

The data obtained in the present study demonstrate that WSPM2.5 induces autocrine AR expression by activating the EGFR-ERK pathway, which is in agreement with a previous study showing that PM2.5 led to granulocyte macrophage colony-stimulating factor (GM-CSF) release by 16HBE human bronchial epithelial cells^29^. The released AR further stimulated AR expression through the EGFR-ERK pathway, thus creating a positive feedback loop between AR and the EGFR-ERK pathway. Because exposure to WSPM2.5 is intermittent, the effects of WSPM2.5 on AR expression and processing could account for the sustained mucin production in exposed populations.

Our study found that AR is closely correlated with increased mucin production. However, AR was found to also be critically involved in the pathogenesis of pulmonary fibrosis^30, 31^. Subjects exposed to biomass smoke have been reported to develop more airway fibrosis than cigarette smokers^32^. Our previous study also indicated that the number of fibroblasts increased significantly in the small airway walls of wood smoke-exposed rats^33^. Thus, the significant airway fibrosis secondary to biomass smoke might be derived from the pro-fibrotic effect of AR. Furthermore, AR is also likely involved in the process of airway smooth muscle remodeling^34^. Further studies are required to address the involvement of AR and EGFR signaling in airway remodeling following WSPM2.5 exposure.

Nonetheless, blocking EGFR with a neutralizing antibody caused the marked accumulation of TGF-α, not AR, in the cell culture supernatant (Supplementary Fig. 2), indicating that TGF-α is rapidly bound by EGFR and utilized by the cells in the absence of the antibody due to its higher affinity for EGFR relative to AR^35^. Moreover, combinations of antibodies against AR, TGF-α and HB-EGF suppressed MUC5AC expression more strongly than an AR-neutralizing antibody alone. This combinatorial effect might again indicate that more than one EGFR ligand contributes to the complex cell response to WSPM2.5.

Our study demonstrated that WSPM2.5 has similar potential to induce MUC5AC production as particles from urban sources^16^, a finding that is in accordance with the conclusion that the adverse pulmonary effects of residential wood combustion appear to be similar to those observed for particles from other sources^13^. Nevertheless, wood smoke contains a complex mixture of many pollutants^36^; thus, we cannot exclude the possibility that other constituents, such as SO_2_ derivatives^37^, leading to MUC5AC induction could have influenced our results. To rule out this possibility, rats will be exposed to PM2.5 alone. The health impacts of other pollutants in woodsmoke, such as carbon monoxide, sulfur oxides and nitrogen, do not change by source as being specific molecules. PM, the most important constituent, however, might have different impacts on health from particles from other sources because of its complex composition. Thus, evaluation of the hazards of PM is pivotal to assess the overall hazard of wood smoke. In addition, these mechanisms were elucidated in the tumor-derived NCI-H292 cell line, which might exhibit physiological properties different from those of primary airway epithelial cells. Additional studies using primary normal human airway epithelial cells are needed to confirm these results.

The average concentration of PM2.5 in the rural kitchen we observed was approximately 4003 μg/m^3^ during episodes of wood use. According to the calculation method of Seriani *et.al*.^38^, if we assume that a man’s hourly ventilation volume is 360 l and the deposition fraction is 20%, approximately 288 μg of PM2.5 is retained in the airway after a 1-h cooking period. Generally, the amount of mucus produced by healthy subjects is approximately 50 ml per day (corresponding to approximately 2 ml per hour). Thus, the concentration of PM in mucus is 144 μg/ml/h, and our *in vitro* dose was low with respect to real-world exposures. Our field measurements recorded peak concentrations of 9833 μg/m^3^ during cooking periods in the kitchen. However, it has been reported that particulate matter could reach 30 mg/m^3^ or more during periods of cooking^39^. Thus, the PM2.5 concentration of 20.4 mg/m^3^ in our study is possible to achieve under certain circumstances.

In summary, this study found that WSPM2.5 induces MUC5AC expression through the AR-EGFR-ERK pathway and that subsequent activation of EGFR-ERK leads to autocrine effects of AR, with the released AR further promoting its own expression. This positive feedback loop might contribute to sustained mucin production in COPD. This study might contribute to a better understanding of the adverse pulmonary effects induced by WSPM2.5. Complete elucidation of the underlying mechanism might provide novel therapeutic targets to reduce mucus overproduction in wood smoke-related COPD patients.

## Materials and Methods

### Materials

Antibodies against phospho-EGFR (Tyr1068), EGFR and MUC5AC (45 M1) were obtained from Abcam (Cambridge, MA, USA). Antibodies against phospho-ERK (Thr202/Tyr204) and ERK, and PD98059 were from Cell Signaling Technology (Danvers, MA, USA). The anti-EGFR neutralizing antibody was from Chemicon (Temecula, CA, USA). The other neutralizing antibodies (anti-TGF-α, anti-AR, and anti-HB-EGF), species- and isotype-matched control antibodies and recombinant AR were from R&D Systems, Inc. (Minneapolis, MN, USA). The GAPDH antibody was from TDYbio (Beijing, China). The MUC5B antibody was obtained from Santa Cruz Biotechnology (Santa Cruz, CA, USA). AG1478, GM6001, TAPI-1 and actinomycin D were purchased from Calbiochem (San Diego, CA, USA). Unless stated otherwise, all other reagents were obtained from Sigma-Aldrich (St. Louis, MO, USA).

## Methods

### Rat handling and chronic exposure to wood smoke

All of the animal experiments were approved by the Local Ethics Committee of Guangzhou Medical University. The detailed process has been described previously^21^. Briefly, 16 nine-week-old female Sprague-Dawley rats (body weights of 180–200 g) were randomly divided into a wood smoke group and a clean air control group. Rats in the wood smoke group were exposed to wood smoke for 1 h four times per day and five days per week for three months. A total of 40 g of China fir sawdust was smoldered, and the smoke generated was channeled through a piston pump to the exposure room. During exposure, the average CO, O_2_ and PM2.5 concentrations in the exposure room were 90.15 ppm, 20.94% and 20.4 mg/m^3^, respectively. The control rats were exposed to clean room air.

### AB-PAS staining and immunohistochemistry

The right lung of each rat was inflated at a 25 cm H_2_O pressure and fixed with 4% paraformaldehyde for 24 h, embedded in paraffin, and cut into 4-μm sections. Para-sagittal sections (n = 5 rats/group) were stained with Alcian blue/periodic acid Schiff (AB-PAS) reagent for mucus detection and immunoblotted with primary antibodies (MUC5AC, 1:50; MUC5B, 1:200) using an immunohistochemistry detection kit (Gene Tech, Shanghai, China) for immunochemistry analysis. Quantitative analysis of lung tissues stained with AB-PAS and various antibodies was completed with Image-Pro plus 6.0 software. The data were calculated using the following equation: % of AB-PAS positively cells = (AB-PAS positive area of airway epithelium)/(total epithelial area) × (the number of airways assessed)^40, 41^. The percentages of MUC5AC and MUC5B positively stained cells were calculated against the total number of cells in the stained area^42^.

### Particle collection and preparation

WSPM2.5 was collected from the burning of China fir in a conventional Chinese wood stove (April 23-May 6, 2015) with a high-volume sampling machine (TE-6070, Tisch, USA) equipped with a PM2.5 selective-inlet head (1.13 m^3^/min). WSPM2.5 was collected on glass fiber membrane filters with a 1.6-μm pore size and a 406-cm^2^ sampling area. Particles were sampled during high-temperature combustion achieved with moderate air supply^43^. The amount of WSPM2.5 was defined as the weight increase for each filter. The filters were soaked in dimethyl sulfoxide (DMSO), and the solutions were then filtered through a 5-μm needle filter. The supernatant was collected, and the particles recovered from different filters were pooled to ensure a homogenous batch of particles. The 40 mg/ml WSPM2.5 stock solution was stored at −20 °C until use. Before treatment, the WSPM2.5 solution was sonicated 3 × 25 s (25 w).

The polycyclic aromatic hydrocarbons (PAHs) and metal content were analyzed via thermal desorption gas chromatography mass spectrometry (TD–GC/MS) and thermal desorption gas chromatography mass spectrometry (TD–GC/MS), respectively, at the Key Laboratory of Aerosol Chemistry and Physics, Chinese Academy of Sciences (XiAn, China) (Supplementary Table 1).

### Cell culture and WSPM2.5 treatment with or without inhibitors

#### Differentiated primary cell culture

Primary human bronchial/tracheal epithelial cells (ATCC, USA) at passage 0 were seeded into 25-cm^2^ flasks in complete bronchial/tracheal epithelial cell growth medium (ATCC) and were passaged when 80% confluent. Cells at passage 3 were seeded in an air-liquid interface at a density of 2 × 10^4^ cells per cm^2^ onto Corning Transwell^®^ inserts pre-coated with collagen I (Corning Costar, USA) and were grown submerged in a 1:1 mixture of epithelial cell basal medium and low-glucose DMEM supplemented with a bronchial epithelial cell growth kit and retinoic acid (50 nM). Once cells were confluent, the apical medium was removed, and the cells were switched to air-liquid interface conditions for two weeks to allow primary cells to differentiate into ciliated, mucus-producing cells. Twenty-four hours before treatment, the cells were grown in medium without growth factors.

#### NCI-H292 cells

NCI-H292 cells were generously provided by Dr. Huahao Shen (The Second Affiliated Hospital, Zhejiang University School of Medicine, China). NCI-H292 cells were cultured in RPMI-1640 medium containing 10% fetal bovine serum. The cells were starved in RPMI-1640 with 0.2% fetal bovine serum to maintain low basal levels of MUC5AC expression for 24 h before experimentation^44^.

#### Cell culture conditions with stimuli and inhibitors

Particles were used at non-cytotoxic concentrations ranging from 0.5 to 48 µg/ml (corresponding to 0.125 to 12 µg/cm^2^) in a dose-response study (Supplementary Fig. 3 **)**, and 8 µg/ml (2 µg/cm^2^) and 40 µg/ml (10 µg/cm^2^) were selected for the subsequent experiments with NCI-H292 cells and primary epithelial cells, respectively. Controls were prepared with extracts from clean filters containing 0.1% DMSO.

Chemical inhibitors or neutralizing antibodies were added 1 h before WSPM2.5 treatment. WSPM2.5, DMSO, neutralizing antibodies and chemical inhibitors were pretested for cytotoxicity using a CCK-8 assay.

### Real-time quantitative PCR

Total RNA was extracted from cells using TRIzol (Invitrogen, USA), and 500 ng RNA was reverse transcribed into cDNA in a final RT reaction volume of 10 µl using the PrimeScript RT Reagent Kit (Takara, Japan). Two microliters of cDNA was amplified in a 25-µl quantitative PCR reaction using SYBR^®^ Premix Ex Taq^TM^ in an MXP3000 QPCR system (Stratagene, USA) under the following conditions: 30 s at 95 °C, 40 cycles of 5 s at 95 °C and 30 s at 60 °C, 1 min at 95 °C and an increase from 60 °C to 95 °C. The primers used are described in Supplementary Table 2. The relative mRNA amounts were calculated using the 2^−△△Ct^ method, and glyceraldehyde-3-phosphate dehydrogenase (GAPDH) was used as an internal control.

### ELISA

The MUC5AC protein expression level in the cell culture supernatants was measured as described by Rada *et al*.^19^, with slight modifications. Briefly, 50 µl of each sample was incubated with bicarbonate-carbonate buffer (50 µl) for 2 h at 37 °C. After the plates were washed three times, they were then blocked with 2% BSA for 1 h and were incubated with 50 µl of mouse anti-MUC5AC antibody (1:200) for 2 h at 37 °C. After the plates were washed three times, 100 µl of horseradish peroxidase-conjugated anti-mouse IgG (1:5000) was added. After 1 h, the plates were washed, and the color reaction was developed with 3, 3′, 5, 5′-tetramethylbenzidine (TMB) peroxidase solution (Thermo Fisher Scientific, USA) and was stopped with 2 M H_2_SO_4_. The absorbance at 450 nm was read. A standard curve was generated with serially diluted samples of bovine submaxillary gland mucin to ensure the linear nature of the colorimetric reaction. The data are expressed as the ratio of the observed value to the control group value.

To analyze EGFR ligand release, ELISA kits for AR, TGF-α and HB-EGF (R&D Systems) were used according the manufacturer’s instructions. The optical density was measured at 450 nm.

### MUC5AC immunofluorescence

Differentiated bronchial/tracheal epithelial cells were fixed with 4% paraformaldehyde for 20 min. After the cells were permeabilized with 0.5% Triton X-100, nonspecific immunoglobulin binding was blocked with 3% BSA for 1 h. A mouse monoclonal MUC5AC antibody (1:100) was incubated at 4 °C overnight. The samples were then incubated with a Cy3-conjugated anti-mouse IgG antibody (1:200) for 1 h. The nuclei were stained with 4′, 6-diamidino-2-phenylindole (DAPI) for 5 min. The inserts were excised, mounted and photographed using confocal microscopy. The percentage of MUC5AC expression was calculated by dividing the MUC5AC fluorescence signal (red) by the total DAPI signal (blue). Fluorescence signals were quantified using Image-Pro plus 6.0 software.

### Protein isolation and Western blot assay

The cells were lysed with RIPA buffer (Pierce, USA) containing protease inhibitor cocktail (Roche, Germany) and phosphatase inhibitor cocktail (Sigma-Aldrich, USA) on ice for 30 min. After centrifugation at 12,000 × g and 4 °C for 20 min, the supernatants were collected. The protein concentrations were determined using the BCA protein assay kit (Pierce, USA). Cell lysates were mixed with 5× SDS-PAGE sample buffer and boiled for 5 min. Thirty micrograms of protein was subjected to 10% SDS-PAGE electrophoresis and transferred to polyvinylidene fluoride membranes. The membranes were blocked with 5% milk and then incubated at 4 °C for 16 h with the following diluted primary antibodies: anti-phospho-ERK1/2 (1:1000) and anti-phospho-EGFR (1:10000). The blots were then washed and probed with horseradish peroxidase-conjugated secondary IgG antibodies. The bound antibodies were visualized using SuperSignal™ West Femto Maximum Sensitivity Substrate (Thermo Fisher Scientific, USA). For normalization, the membranes were stripped with Restore Western blot stripping buffer and incubated with the following primary antibodies: anti-ERK (1:2000), anti-EGFR (1:100,000), and anti-GAPDH (1:1000).

### Statistical analysis

The data are expressed as the means ± SD from at least three independent experiments. One-way ANOVA followed by Tukey’s multiple comparison test was applied to assess the differences between multiple groups. Differences between two groups were analyzed using an unpaired *t* test. *P* values < 0.05 were considered statistically significant.

## Electronic supplementary material

supplementary
