# Supplementary material for: Positive feedback of the amphiregulin-EGFR-ERK pathway mediates PM2.5 from wood smoke-induced MUC5AC expression in epithelial cells
Source: Sci Rep. 2017 Sep 11;7:11084. doi: 10.1038/s41598-017-11541-1 (PMC5593934; doi:10.1038/s41598-017-11541-1)
Supplement: Supplementary file 1 — supplementary [file 41598_2017_11541_MOESM1_ESM.pdf]

Positive feedback of the amphiregulin-EGFR-ERK pathway mediates PM2.5 from wood smoke -induced MUC5AC expression in epithelial cells

Lingmei Huang[1, 4], Jinding Pu[1], Fang He[2], Baoling Liao[1], Binwei Hao[1], Wei Hong[1, 3], Xiuqin Ye[1], Jinglong Chen[1,5], Jun Zhao[1,5], Sha Liu[1], Juan Xu [1], Bing Li[3*], Pixin Ran[1]*

Contents:

- **Supplementary Figure 1.** Effect of NAC on the phosphorylation of EGFR-ERK signaling.
- **Supplementary Figure 2.** The effect of EGFR-ERK pathway on TGF-α level.
- **Supplementary Figure 3.**Effects of WSPM2.5 on Cell Viability.
- **Supplementary Table 1.** Polycyclic aromatic hydrocarbons (PAHs) and metal content in WSPM2.5
- **Supplementary Table 2.** Real-Time PCR Primers.

Supplementary Figure 1. Effect of NAC on the phosphorylation of EGFR-ERK signaling.

N-acetyl-L-cysteine (NAC), a common antioxidant, was used to assess the effect of reactive oxygen species on the phosphorylation of EGFR and ERK. Treatment with the NAC significantly reduced EGFR and ERK phosphorylation (Fig. 1).

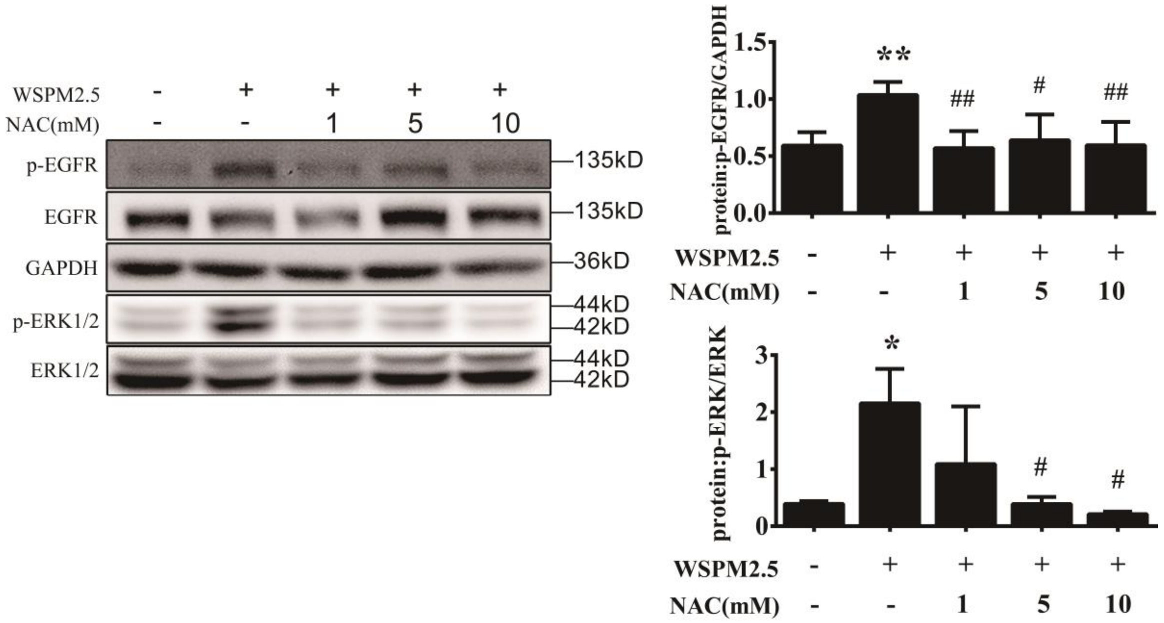

Fig. 1. Reactive oxygen species mediates activation of EGFR-ERK pathway. NCI-H292 cells were pretreated with or without NAC (1, 5, 10 mM) for 1 h, then were stimulated with WSPM2.5 (8 μg/ml) for 60 min or 5 min to access the phosphorylation of EGFR or ERK, respectively. One representative gel from five independent experiments is shown. Densitometry analysis of p-EGFR was performed after normalization to GAPDH. Data are expressed as the mean ± SD (n = 5). *, $P < 0.05$, **, $P < 0.01$ compared with control; #, $P < 0.05$, ##, $P < 0.01$ compared with the WSPM2.5 group.

Supplementary Figure 2. The effect of EGFR-ERK pathway on TGF-α level.

A small but without significant significance increase was detected in TGF-α release in response to WSPM2.5. However, the EGFR-neutralizing antibody caused a marked increase of TGF-α compared with WSPM2.5 treatment alone, indicating that TGF-α is

rapidly bound to the receptor and utilized by the cells in the absence of anti- EGFR antibody. Both AG1478 and PD98059 led to a significantly reduction of TGF-α levels.

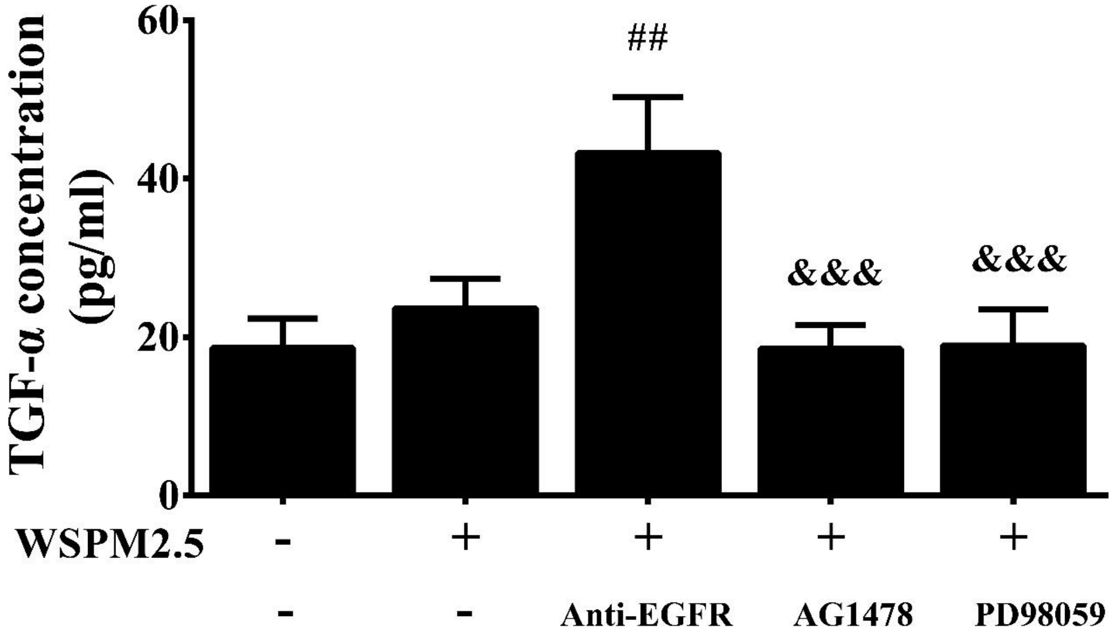

Fig. 2. Release of TGF-α from NCI-H292 cells. NCI-H292 cells were pretreated with or without EGFR neutralizing antibody (0.5 μg/ml), AG1478 (10 μM) or PD98059 (5 μM) for 1 h and then stimulated with WSPM2.5 for 24 h. TGF-α level was assessed by ELISA. Data are expressed as the mean ± SD; n = 3. ###, $P < 0.001$ compared with WSPM2.5-treated cells. &&&, $P < 0.001$ compared with Anti-EGFR group.

Supplementary Figure 3. Effects of WSPM2.5 on Cell Viability.

The toxicity of WSPM2.5 for NCIH292 cells way assessed by CCK-8 assay. The effect of WSPM2.5 was dose-dependent: at lower concentrations (0-24 μg/ml), cell survival was enhanced; however, at concentrations of 48 μg/ml and above, WSPM2.5 was toxic to cells. The effects were similar at the 24 h, 36 h and 48 h time points. At different time points, cell viability all increased significantly at 8 μg/ml.

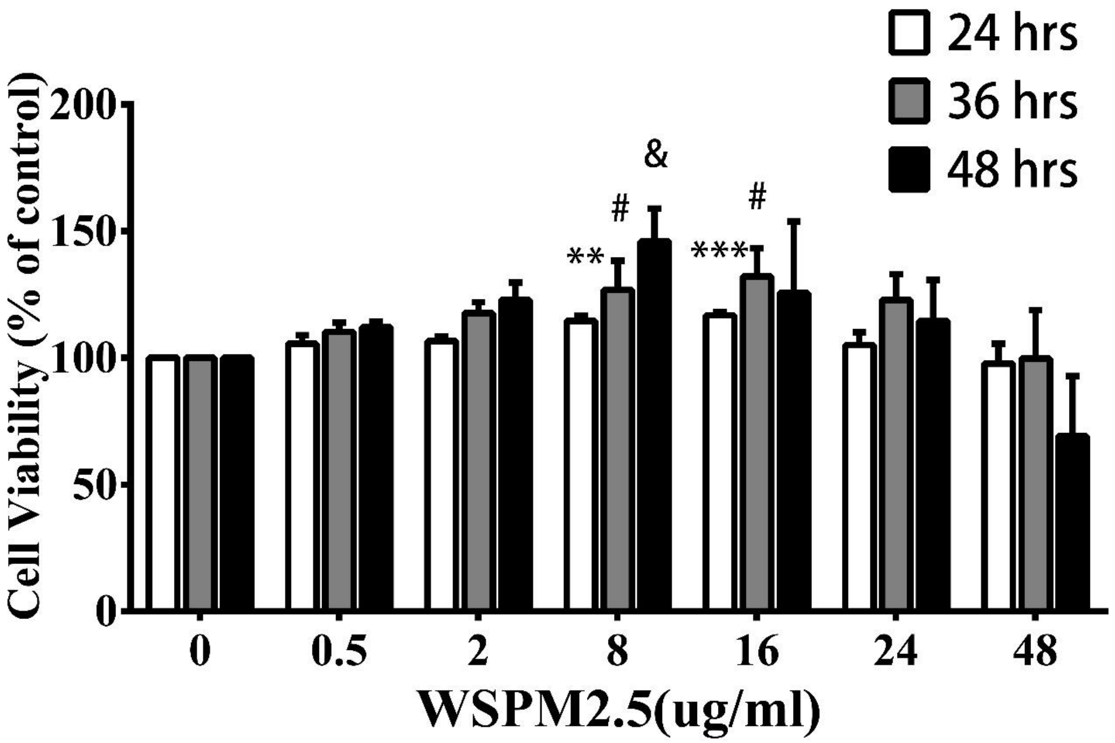

Fig. 3. The effects of WSPM2.5 on NCIH292 cells viability. Cells were stimulated with WSPM2.5 for 24, 36 or 48 h. Cell viability was determined by CCK-8 assay. Values are expressed as the mean ± SD; n = 3. **, $P < 0.01$ compared with control at 24 h. ***, $P < 0.001$ compared with control at 24 h. #, $P < 0.05$ compared with control at 36 h. &, $P < 0.05$ compared with control at 48 h.

Tables

Table. 1. Polycyclic aromatic hydrocarbons (PAHs) and metal content in WSPM2.5

**PAHs（μg/g PM2.5）**	**Concentration**
Naphtalene	0.682
Acenaphthene	0.673
Acenaphthene	N.D.
Fluorene	N.D.
Phenanthrene	32.652
Anthracene	1.144
Fluoranthene	62.824
Pyrene	59.1035
Benzo[a]anthracene	287.664
Chrysene	83.885
Benzo[b]fluoranthene	151.653
Benzo[k]fluoranthene	N.D.
Benzo[a]fluoranthene	15.857
Benzo[e]pyrene	58.046
Indeno[1,2,3-cd]pyrene	70.7495
Dibenzo[a,h]anthracene	73.021
Benzo[ghi]perylene	16.3945
∑Total	914.0115
**Metals(mg/g PM2.5）**	
S	0.184
K	44.815
Cl	135.34
Si	N.D.
Fe	0.019
Na	5.534
Zn	0.174
Ca	0.129
Al	N.D.
Pb	0.401
Cu	0.034
Ba	0.215
Mn	N.D.
Ti	0.014
Mg	N.D.
Sb	0.05

V	N.D.
Cr	N.D.
Rb	0.202
Co	N.D.
Ni	N.D.
∑Total	186.907

N.D.: not detected.

Table. 2. Real-Time PCR Primers.

Gene	**Real-Time PCR Primers.**		Length (bp)
Rat Muc5ac	Forward	5'-GTA ACC AGA CGG GCT GTG TC-3'	120
	Reverse	5'-TCC ACT GAC CTC CAG AGC AC-3'	
Rat Muc5b	Forward	5'-GGA CCA GAG GTG GGA AAG AG-3'	141
	Reverse	5'-TAG GGT TGG ACA AGG GGA GG-3'	
Rat GAPDH	Forward	5'-GGT GAT GCT GGT GCT GAG TAT G-3'	153
	Reverse	5'- CTC GTG GTT CAC ACC CAT CAC-3'	
Human MUC5AC	Forward	5'-ACG TGT TCT CCG AGC ACT GC-3'	201
	Reverse	5'-GCT GAA TGA GGA CCC CAG AC-3'	
Human AR[1]	Forward	5'-TGG TGC TGT CGC TCT TGA TA-3'	149
	Reverse	5'-CCC TGA AGA CAT CTC ACT TC-3'	
Human TGF-α[1]	Forward	5'- CCC TGG CTG TCC TTA TCA TC-3'	145
	Reverse	5'- GTT TCT GAG TGG CAG CAA GC-3'	
Human HB-EGF[1	Forward	5'- GGA GAG GAG GTT ATG ATG TG-3'	248
	Reverse	5'-TTT GGC ACT TGA AGG CTC TG-3'	
Human GAPDH	Forward	5'-CAG CCT CAA GAT CAT CAG CA-3'	138
	Reverse	5'-ACA GTC TTC TGG GTG GCA GT-3'	

References:

1. Rumelhard, M., Ramgolam, K., Hamel, R., Marano, F. & Baeza-Squiban, A. Expression and Role of EGFR Ligands Induced in Airway Cells by PM2.5 and its Components. *EUR RESPIR J*. 30, 1064-1073 (2007).
